# Live-cell imaging unveils distinct R-loop populations with heterogeneous dynamics

**DOI:** 10.1093/nar/gkad812

**Published:** 2023-10-11

**Authors:** Robert M Martin, Madalena R de Almeida, Eduardo Gameiro, Sérgio F de Almeida

**Affiliations:** Instituto de Medicina Molecular João Lobo Antunes, Faculdade de Medicina da Universidade de Lisboa, Lisboa, Portugal; Instituto de Medicina Molecular João Lobo Antunes, Faculdade de Medicina da Universidade de Lisboa, Lisboa, Portugal; Instituto de Medicina Molecular João Lobo Antunes, Faculdade de Medicina da Universidade de Lisboa, Lisboa, Portugal; Instituto de Medicina Molecular João Lobo Antunes, Faculdade de Medicina da Universidade de Lisboa, Lisboa, Portugal

## Abstract

We have developed RHINO, a genetically encoded sensor that selectively binds RNA:DNA hybrids enabling live-cell imaging of cellular R-loops. RHINO comprises a tandem array of three copies of the RNA:DNA hybrid binding domain of human RNase H1 connected by optimized linker segments and fused to a fluorescent protein. This tool allows the measurement of R-loop abundance and dynamics in live cells with high specificity and sensitivity. Using RHINO, we provide a kinetic framework for R-loops at nucleoli, telomeres and protein-coding genes. Our findings demonstrate that R-loop dynamics vary significantly across these regions, potentially reflecting the distinct roles R-loops play in different chromosomal contexts. RHINO is a powerful tool for investigating the role of R-loops in cellular processes and their contribution to disease development and progression.

## Graphical Abstract

**Graphical Abstract:**
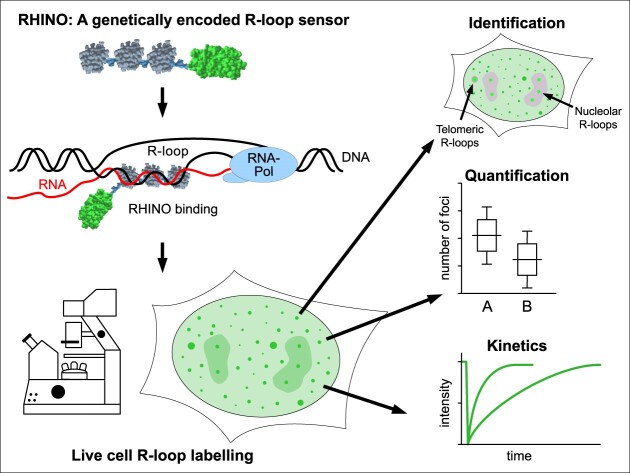

## Introduction

R-loops are non-canonical nucleic acid structures composed of an RNA:DNA hybrid and a displaced DNA strand, which play roles in numerous cellular processes, but are also an important source of genomic instability (1,2). They usually form during transcription when a nascent RNA molecule hybridizes with the template DNA strand. Despite known issues and limitations, such as binding to double-stranded RNA (dsRNA) and the inability to profile live cells, the S9.6 antibody that recognizes RNA:DNA hybrids is still the main tool used for R-loop detection (3). Other commonly used methods to map and quantify R-loops exploit the RNase H1 enzyme, which degrades the RNA moiety of the R-loops restoring the double-stranded conformation of the two DNA strands (4). The enzymatic specificity towards RNA:DNA hybrids is conferred by a hybrid binding domain (HBD). The catalytically-dead version of RNase H1 and the isolated HBD have been employed as R-loop detection tools in imaging and immunoprecipitation-based assays, yet their capacity to provide accurate measurements of R-loop dynamics in live cells has not been sufficiently demonstrated (3–6).

In this study, we sought to create a new tool suitable for live-cell imaging of R-loops. We developed RHINO, a genetically encoded **R**NA:DNA **h**ybrid-b**in**ding sens**o**r that detects R-loops *in vivo* and can be used to gather novel insights into R-loop dynamics at distinct genomic locations. The high R-loop-detection specificity of RHINO allows for accurate R-loop imaging and quantification of kinetic parameters. Here, we measured the dynamics of R-loops at three different loci: nucleoli, telomeres and protein-coding genes. Our data reveal the heterogeneity of R-loop dynamics, which define distinct R-loop populations.

## Materials and methods

### Plasmids and genetic constructs

Restriction and DNA modification enzymes used for cloning were purchased from Thermo-Fisher unless otherwise noted. The original construct with a GFP-tagged hybrid binding domain (HBD) containing 50 amino acids of the N-terminus of the short M27 protein variant from human RNase H1 was first described by Bhatia *et al.* (7). Based on this, RHINO was de novo designed to consist of three head-to-tail tandem HBDs with neutral linker sequences and constructed by a first g-Block (IDT) containing the N-terminal FLAG and V5 tags followed by a single HBD sequence which was ligated into a pUC vector for amplification. Next, a second g-block containing a neutral N-terminal linker sequence, followed by a single HBD sequence with an M to V (ATG to GTG) substitution at the first amino acid position was designed and ligated into a pEGFP-C1 vector for amplification. A BglII + BamHI fragment containing the N-terminal linker followed by the HBD sequence lacking the ATG(Met) codon was sequentially ligated into pUC-FLAG-V5-HBD to generate three tandem HBDs. Next, an oligo encoding a linker sequence with a frameshift was ligated into the BamHI site downstream of the last HBD sequence in pUC-FLAG-V5-3xHBD.

The NheI + BamHI fragment containing the FLAG-V5-3xHBD-linker sequence was then subcloned into a pUBC-mNeonGreen-N1 vector to generate the final pUBC-FLAG-V5-3HBD-mNeonGreen (RHINO).

The pUBC-mNeonGreen-N1 was constructed by PCR amplification of the mNeonGreen fluorescent protein from mNeonGreen-mTurquoise2 (mNeonGreen-mTurquoise2 was a gift from Dorus Gadella; Addgene plasmid #98886; http://n2t.net/addgene:98886; RRID:Addgene_98886) using the NZYProof DNA polymerase (NZYTech, Lisbon Portugal). The mNeonGreen PCR fragment was then ligated into pEGFP-N1 replacing EGFP. A fragment containing the N-terminal multiple cloning site followed by mNeonGreen was then subcloned into the pUBC vector described before (8).

The pUBC-mScarlet-i-RNase H1 (WT) and (D210N) constructs were generated by PCR amplification of the human RNase H1 short (nuclear) protein version from ppyCAG_RNase H1_WT and ppyCAG_RNase H1_D210N, respectively. ppyCAG_RNase H1_WT and ppyCAG_RNase H1_D210N were gifts from Xiang Dong Fu, Addgene plasmid #111906 and Addgene plasmid #111904. The PCR products were then each ligated into pUBC-mScarlet-i-C1, derived by replacing GFP in pUBC-GFPC1 with mScarlet-I from pmScarlet-i_C1 (pmScarlet-i_C1 was a gift from Dorus Gadella, Addgene plasmid # 85044; http://n2t.net/addgene:85044; RRID: Addgene_85044).

The pCMV-GFP-1HBD (7) construct was subcloned by ligating the BglII + BamHI fragment containing one HBD into the BamHI site in pUBC-GFP-C1 vector under the control of the ubiquitin C promoter.

The pL30-mCherry-C1 plasmid was made by ligating a SmaI + Klenow blunted XagI fragment from pL30-GFP-MS2 (a kind gift from Edouard Bertrand (9)) and ligating it into the blunted VspI + BspEI sites in pEGFP-C1 thereby replacing the CMV promoter and the GFP sequence. Next, a BamHI + HindIII fragment from pL30-mCherry-MS2 (a kind gift from Edouard Bertrand (9)) containing the mCherry sequence was ligated into the same sites of the pL30-C1 backbone. The SV40 NLS was ligated into the Bsp1407I site using complementary oligos to generate pL30-mCherry-NLS-C1.

The pL30-mCherry-H2B plasmid was constructed by obtaining the histone H2B sequence from pH2B-mRFP (10) by BglII + BamHI digest and ligating into the BamHI site of pL30-mCherry-C1 (8). The pmCherry-nls-8R plasmid was generated by ligating a double-stranded oligo into the BglII site of pL30-mCherry-C1 plasmid. The oligo encoding an SV40 nuclear localization sequence (NLS) followed by 8 Arginine (11,12) was prepared as follows: complementary single-stranded DNA primers were diluted to 10 μM, 2 μl of each primer were mixed in annealing buffer (10 mM Tris–HCl, 50 mM NaCl). Annealing was performed by heating to 95°C followed by cooling at 0.1°C/s to 4°C in a PCR machine. The annealed oligos were phosphorylated using T4-PNK (Thermo-Fisher) in a standard reaction.

A pUBC-iRFP-C1 vector was constructed by amplifying iRFP713 from pIRES-H2B-iRFP (a kind gift from Edgar Gomes) by PCR with primers containing 5′ BshTI and 3′ BglII sites. The digested PCR product was ligated into the same site in pUBC-GFP-C1 replacing the EGFP sequence. The pUBC-iRFP-TRF2 construct was cloned by cutting the human TRF2 sequence from pLVC-Nmyc-hTRF2 (a kind gift from Claus M. Azzalin) with BamHI + NotI and ligating it into the BglII and Bsp120I site in pUBC-iRFP-C1.

The pL30-mCherry-NLS-rtTA was generated by PCR amplification of the rtTA sequence from pLVX-TetONE-Puro (a kind gift from Claus M. Azzalin) using primers with BglII and BamHI extensions. The digested PCR product was then ligated into the same sites in a pL30-mCherry-NLS-C1 vector.

The RNase H1-NES-GR-iRFP construct was made by replacing I-SceI with RNase H1 in the pI-SceI-GR-iRFP construct described by our lab before (8). The RNase H1 was PCR amplified from the plasmid pICE-RNase HI-WT-NLS-mCherry (pICE-RNase HI-WT-NLS-mCherry was a gift from Patrick Calsou, Addgene plasmid #60365; http://n2t.net/addgene:60365; RRID: Addgene_60365), with primers with BshTI and Acc65I extensions using the NZYProof DNA polymerase (NZYTech, Lisbon Portugal). The purified PCR product was cut with BshTI and Acc65I and ligated into the same sites. To retain the construct in the cytoplasm before adding TA, a nuclear export sequence (NES) from the heat-stable inhibitor (PKl) of cAPK (13) was added in frame by ligating a double-stranded oligonucleotide with compatible overhangs into the Acc65I site. The NES oligonucleotide was prepared according to the protocol described above.

The IgM reporter gene was constructed by first ligating a blunted XhoI + BshTI TRE3GS containing fragment from pLVX-TetONE-Puro into the blunted BglII + BshTI sites in pcDNA5/FRT/TO (Thermo Fisher Scientific/Invitrogen) to replace the CMV promoter and introduce the TRE3GS promoter with 7x TetO repeats (Takara Bio Inc.). Next, complementary oligos encoding 2× TetO repeats were serial ligated into the SgrDI (NEB) site until a total of 13× TetO repeats. The 24xPP7 stem-loop array was obtained from pCR4-24xPP7SL (pCR4-24XPP7SL was a gift from Robert Singer; Addgene plasmid # 31864; http://n2t.net/addgene:31864; RRID: Addgene_31864) (14) by BglII and BamHI digest followed by Klenow blunting and ligating into the Klenow blunted NotI site of the aforementioned reporter gene construct. A BspTI and XhoI fragment containing the complete reporter construct was ligated into the same sites in p13TO-TRE3GS-FRT.

The shRNA expression plasmid was cloned by fusing a PCR-amplified Puromycin resistance gene in frame to iRFP713 and ligating the resulting construct into a pSuper-Puro construct, thereby replacing Puro with iRFP-Puro. The H1 promoter was replaced by a PCR amplified U6 promoter fragment for shRNA expression. This construct served for the selection of shRNA expression vector-transfected cells. The shRNA sequences were cloned downstream of the U6 promoter into BglII and SalI sites. The *DDX23* shRNA was designed using the Broad Institute Genetic Perturbation Platform (GPP) online tool. The target sequence and the homologous antiparallel sequence were separated with the miR22 loop sequence and followed by 6 thymidines as terminators. The control GL2 target sequence was derived from Addgene plasmid #86083 (15).

### Cell lines

The U2OS, U2OS-bWT#9, U2OS Flp-in T-REx and HeLa cell lines were grown as monolayers in high-glucose (4.5 g/l) Dulbecco's modified Eagle's medium (DMEM) supplemented with 10% (v/v) fetal bovine serum (Gibco) and 1% (v/v) l-glutamine (Thermo Fisher Scientific). As a parental cell line to receive and express the IgM reporter-gene constructs, we used U2OS Flp-in T-REx cells (a kind gift from Karmella Haynes) (16). Isogenic stable cell lines were generated through Flp recombinase-mediated integration by co-transfecting the plasmid pOG44 expressing the Flp recombinase (Invitrogen/Thermo Fisher Scientific) and the p13TO-TRE3GS-FRT vector containing the IgM reporter gene constructs. After transfection, the U2OS Flp-in T-Rex cells were maintained under selective pressure in the presence of 200 mg/ml Hygromycin B (InvivoGen) and 15 mg/ml Blasticidin (InvivoGen).

### Live cell experiments

For live cell imaging experiments, the cell lines were seeded in DMEM with HEPES, without phenol red (Gibco) supplemented with 10% (v/v) fetal bovine serum (Gibco) and 1% (v/v l-glutamine (Thermo Fisher Scientific). For the live cell imaging experiments, cells were plated on 35-mm Petri dishes with 10 or 14-mm glass-bottom microwell (coverglass thickness no. 1.5) (MatTek Corporation). Cells were transiently transfected with one or more plasmids simultaneously 16 to 24 hours before experiments using Lipofectamine 3000 reagent (Thermo Fisher Scientific), according to the manufacturer's protocol. Transcription of the reporter genes was induced with doxycycline (0.5 μg/ml; D9891, Sigma-Aldrich), where indicated, and nuclear translocation of pRNase H1–NES-GR-iRFP or was induced by adding TA (T6501, Sigma-Aldrich), prepared in dimethyl sulfoxide (DMSO), and, at the time of treatment, diluted in the abovementioned culture medium (final concentration 10^−7^ M). A 1 M Triptolide stock solution was prepared in DMSO and diluted in the abovementioned culture medium (final concentration 1 μM). The 5,6-Dichlorobenzimidazole 1-β-d-ribofuranoside (DRB) (D1916, Sigma-Aldrich, Merck) was dissolved in DMSO to a 50 mM stock solution and diluted in culture medium to a final concentration of 100μM. Triptolide, TA and DRB addition to live cells grown in microscopy dishes was performed as described before (17).

### Immunofluorescence

Cells were seeded on glass coverslips No.1 (Marienfeld, Germany) in 6 well plates and subsequently transfected as described. The next day, the growth medium was removed and cells were briefly washed in pre-warmed 1× PBS and fixed for 10 min with 3.5% Formaldehyde in 1× PBS. After removing the fixative, cells were washed twice with 1× PBS and permeabilized with 0.5% Triton X-100 in 1× PBS for 10 min, followed by two washing steps in 1× PBS and incubation with a mouse-anti-UBF antibody (sc-13125, Santa Cruz) at 1:200 dilution in antibody diluting solution (0.05% Triton X, 0.1% Sodium azide, 0.2% fish skin gelatine in 1X PBS) for 1 h at 37°C in a humidified chamber. After removing the primary antibody solution, cells were washed briefly twice in 1× PBS-Tween, then incubated with a secondary Cy3 conjugated goat anti-mouse antibody (A90-516C3, Bethyl) at 1:200 dilution in 1× PBS–Tween for 1 h at 37°C in a humidified chamber. For the γH2AX staining, formaldehyde-fixed cells on coverslips were incubated with a mouse-anti-phospho-Histone H2AX (Ser139 antibody 05–636, clone JBW301, Merck) followed by incubation with a goat-anti-mouse antibody conjugated with Alexa647 (A-21236, ThermoFisher). For R-loops antibody staining, cells were fixed with methanol for 10 min at –20°C, washed twice with PBS 1x and incubated with a mouse anti-DNA:RNA hybrid antibody (MABE1095, clone S9.6, Merck) at a 1:100 dilution in antibody diluting solution for 1h at 37°C in a humidified chamber. After removing the primary antibody solution, cells were washed briefly twice in 1× PBS–Tween, then incubated with a secondary Cy3-conjugated goat anti-mouse antibody (A90-516C3, Bethyl) at a 1:200 dilution in 1× PBS–Tween for 1 h at 37°C in a humidified chamber. Coverslips were then washed twice with 1× PBS–Tween, incubated with 1 μg/ml DAPI in 1× PBS for 10 min at 37°C and again washed twice with 1× PBS–Tween. The coverslips were next briefly dipped in ddH_2_O, excess water removed and mounted in Fluoromount-G (Invitrogen/ThermoFisher) on glass microscopy slides (Normax, Portugal).

### Microscopy

All microscopy experiments were performed at the Bioimaging Unit of Instituto de Medicina Molecular—João Lobo Antunes. Live cell imaging was performed on a 3i Marianas SDC spinning disk confocal imaging system (Intelligent Imaging Innovations Inc.) using a similar microscopy setup previously described (34). The system is based on an Axio Observer Z1 inverted microscope (Carl Zeiss MicroImaging Inc., Germany) equipped with a Yokogawa CSU-X1 spinning disk confocal head (Yokogawa Electric, Tokyo, Japan) and 100-mW solid-state lasers (Coherent Inc., Santa Clara, CA) coupled to an acoustic-optic tunable filter. The axial position of the sample was controlled with a piezo-driven stage (Applied Scientific Instrumentation, Eugene, OR). Each MatTek dish was placed in an incubation chamber (PeCon P-Set 2000, PeCon GmbH, Erbach, Germany) mounted on the microscope stage and connected to CO_2_ (CO_2_ module S, PeCon) and humidity (Heating Device Humidity 2000, PeCon) controllers. The whole microscope body excluding lasers, camera and spinning disk head was maintained inside a large plexiglass environmental chamber (PeCon, Erbach, Germany). The temperature in both the microscope and top-stage incubation chambers was controlled by a common unit and set to 37°C. The environment inside the top stage incubation chamber was further set to 5% CO_2_ and 100% humidity. Samples were illuminated with l = 488 nm for GFP/mNeonGreen, l = 561 nm for mCherry and l = 640 nm for iRFP713. Images were acquired using a 100× (Plan Apo, 1.4 numerical aperture) oil immersion objectives (Carl Zeiss MicroImaging Inc.) under the control of SlideBook 6.0 software (Intelligent Imaging Innovations, Denver, CO). Three-dimensional (3D) image stacks of 30 to 40 optical slices separated by 0.32 μm were collected, with exposure acquisition times of 50 ms. Digital images (16-bit) were acquired using a back-thinned air-cooled electron-multiplying charge-coupled device camera (Evolve 512, Photometrics, Tucson, AZ). To select cells with a similar expression level of RHINO for image acquisition, the camera scale image display was set to constant limits of 450–3000.

Additional immunofluorescence and live cell images were acquired on an LSM 880 laser scanning confocal microscope mounted on an Axiovert microscope body (Carl-Zeiss MicroImaging Inc., Germany) and equipped with a large cage and stage stop incubators (Pecon, Erbach, Germany) maintained at 37°C and 5% CO_2_ with humidification. Images were acquired using a Plan-Apochromat 63×/1.4 oil immersion objective. DAPI and Hoechst fluorescence was detected using 405 nm for excitation (Diode laser with 30 mW nominal output – 2% transmission) and a 415–475 nm detection window. GFP/mNeonGreen fluorescence was detected using the 488 nm laser line of an Ar laser for excitation (25 mW nominal output—1–3% transmission) and a 498–557 nm detection window, using the GaAsP detector. mCherry/Cy3 fluorescence was detected using 561 nm for excitation (HeNe laser with 2 mW nominal output—5% transmission) and a 600–735 nm detection window, with PMT gain set to 700 and offset to 1. The pinhole size was set to accommodate 0.7 μm confocal sections for each channel. Z-stacks of the three channels were acquired with zoom 5 (1024 × 1024 pixel frame size—0.026 μm pixel size) at 0.35 μm slice intervals covering the complete nuclear volume. The FRAP experiments were performed on the LSM 880 system with the described characteristics. To perform the bleaching, a circular region of interest (ROI) with a 25-pixel diameter at zoom 5 was exposed to 100% intensity of the 488 nm laser for two iterations at 8.24 μsec pixel dwell time. The time-lapse imaging was performed by acquiring 5 pre-bleach and 120 post-bleach images at 300 ms time intervals of a square region of 128 × 512 pixels.

Super-resolution imaging of RHINO labelling was performed on a VT-iSIM multipoint scanner system (VisiTech International Ltd, Sunderland, UK) mounted on a Nikon Ti-2-E inverted microscope with Perfect Focus System which is housed in an Okolab OKO cage incubation system equipped with a stage incubation chamber (Okolab, Ottaviano, Italy). Images were acquired using a Nikon CFI SR Apochromat TIRF 100× AC Oil objective and an additional 1.5× magnifying lens (Nikon Europe B.V.) with a Prime BSI Express sCMOS Camera (Photometrics). The fluorophores were excited by solid-state lasers L488P150 – 488 nm (150 mW) for mNeonGreen, L561P100 – 561 nm (100 mW) for mCherry and L642P100 – 642 nm (100 mW) for iRFP713. The Z-stack acquisition was done at 0.2 μm spacing and image post-processing was performed with the Richardson-Lucy algorithm for structured illumination deconvolution.

Additional super-resolution images were acquired using a Zeiss LSM 980 laser scanning confocal microscope using a 63×/1.4 oil immersion objective and the Airyscan 2 detector in SR (super-resolution) mode. DAPI fluorescence was detected using 405 nm for excitation (15 mW nominal output – 2% transmission) and a 422–477 nm + 573–627 nm dual bandpass emission filter, with the gain set to 700 V and offset to 0. The mNeonGreen fluorescence was detected using 488 nm for excitation (13 mW nominal output – 1.0% transmission) and a 499–557 nm + 659–735 nm dual bandpass emission filter, with the gain set to 737 V and offset to 0. Cy3 fluorescence was detected using 561 nm for excitation (13 mW nominal output – 0.4% transmission) and a 573–620 nm + 655–735 nm dual bandpass filter, with the gain set to 728 and offset to 0. Z-stacks of the three channels were acquired with Zoom set to 3.4 (39.61 × 39.61 μm area with 1122 × 1122 pixel frame size - 0.035 μm pixel size corresponding to 2x Nyquist) over a range of 5.46 μm (40 slices, 0.140 μm slice interval), with a line average of 1 and 3.74 μs pixel dwell time (bidirectional scan). The image post-processing was performed using the Joint-Deconvolution algorithm of the ZEN 3.5 (blue edition) software package.

### Image analysis

Image stacks were analyzed in the ICY image analysis package (18). To measure the number and intensity of RHINO-labelled foci in 3D, the Spot Detector plug-in was used with the following settings. Images were pre-processed using the ‘Mean Of All Channels’. For spot detection, the UDWT Wavelet Detector was used to detect bright spots over dark backgrounds enforcing the use of 2D Wavelets over 3D. Scale sensitivity was set to Scale 1 (1 pixel) = disabled, Scale 2 (3 pixels) = 60, Scale 3 (7 pixels) = 130. A size filtering was applied using a minimal size of 2 and a maximum size of 50. To restrict spot detection to the cell nuclei, polygon ROIs were drawn around the nuclei according to the RHINO fluorescence accumulation. Spots were detected in 3D and those spots located outside the nuclear perimeter in the x-, y- and z-direction were removed. Data collection, analysis and statistics were done in Excel and OriginPro.

To measure the fraction of telomeres with RHINO signals above the diffuse background fluorescence intensity, merged image stacks were analyzed with the ICY image analysis package to segment the telomere signals in 3D and register the regions of interest. The mean RHINO fluorescence intensity was measured in the regions of interest for the RHINO channel. The mean RHINO fluorescence intensity was also measured in a nucleoplasmic region with diffuse background RHINO distribution. The mean RHINO fluorescence intensity for each telomere was compared to the mean RHINO background intensity value of the same cell to calculate the number of telomeres with RHINO signal accumulation.

The quantification of γH2AX foci was done using the FIJI software package employing a macro to segment the nuclei according to the DAPI staining and count the γH2AX foci above a size threshold of 3 pixels in a maximum intensity projection from the 3D image stack inside the pre-determined boundary of the nucleus.

The FRAP time-lapse imaging was analyzed using the FIJI software package (19). The analyses of the FRAP curves were based on a method described before (20). Time-lapse images were aligned using the TurboReg plugin for FIJI was used (21). From the raw data, three measurements were obtained for each time point: the mean fluorescence intensity in the bleached ROI; the mean background fluorescence intensity in a region outside the cell lacking significant fluorescence signals; and the decrease in the overall fluorescence intensity of the RHINO labelled nucleus excluding the bleach ROI to compensate for the loss of fluorescence due to continuous imaging. The Data were normalized in Excel by defining the mean fluorescence intensity of the ROI before bleaching to 1.0 and the intensity in the same ROI in the first post-bleach image to 0. RHINO intensity in 3D time series imaging experiments was measured using the STaQTool software as described before (22). The line intensity profiles were measured using the FIJI software package.

### Statistics

For RHINO foci quantification in random cells in a population (Triptolide treatment, DRB treatment and RNase H1 overexpression) and for the gH2A.X foci quantification, a non-parametric Mann–Whitney *U* test was applied. A paired sample *t*-test was applied for the same cells quantified for RHINO foci before and after treatment with Triptolide and RNase H1-NES-GR-iRFP. A Student's t-test was applied to FRAP curves approaching plateau values and a 2-sample *t*-test was applied to the different RHINO FRAP fractions at telomeres and the RHINO fluorescence intensity measurements at the β-globin reporter gene upon DRB treatment. A p-value < 0.05 was chosen as the limit of significance and marked as *P < 0.05, **P < 0.01, ***P < 0.001. The quantification of RHINO foci was presented as scatter plots with overlaid box plots. The box plots display the two centre quartiles with arithmetic means ± standard deviations.

## Results

### A genetically encoded sensor for live-cell imaging of R-loops

**Figure 1.**
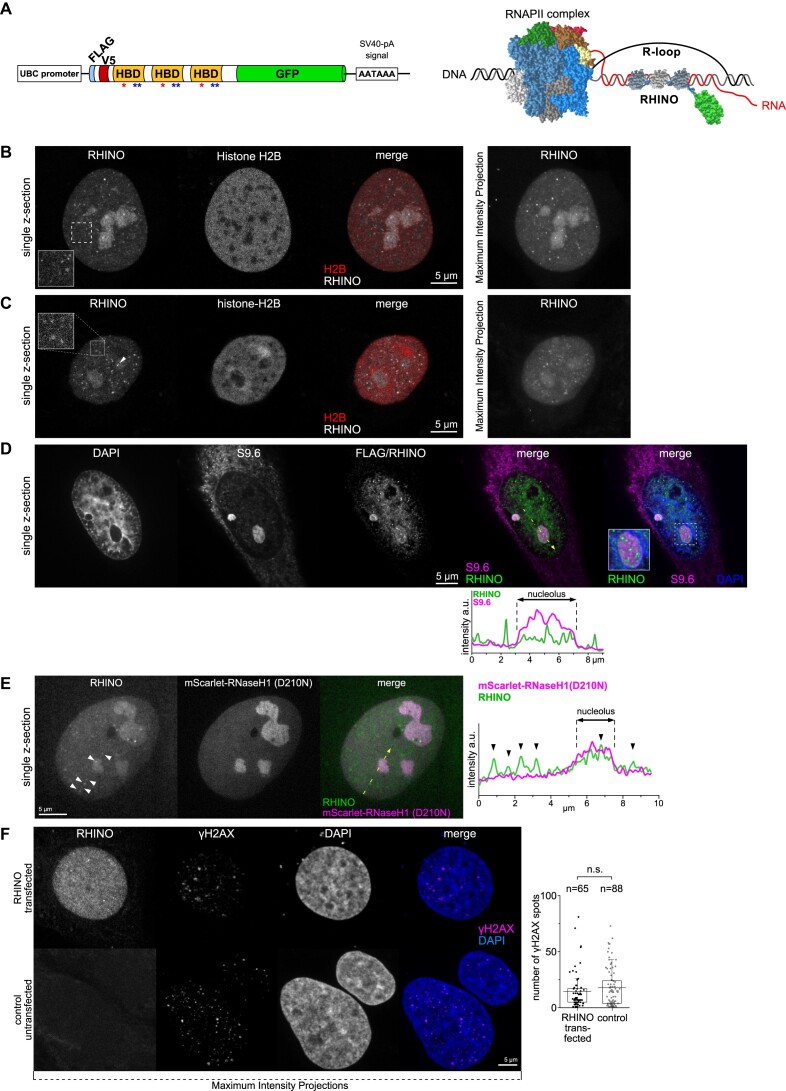
Live-cell imaging of R-loops. (**A**) Illustration of RHINO structure (left) and R-loop labelling (right). The positions of the amino acids substituted in the RHINO mutants are indicated by red (W43A) and blue (KK59/60AA) asterisks. Live-cell imaging of R-loops (using RHINO) and histone H2B by laser scanning confocal microscopy in U2OS (**B**) and HEK293 (**C**) cells. The images show single z-sections from confocal 3D z-stacks covering the entire nuclear volume and the corresponding maximum intensity projection. Note the RHINO spots throughout the nucleoplasm (insets). (**D**) Comparison of RHINO and S9.6 antibody labelling of R-loops in methanol-fixed U2OS cells. The nucleolar signals of S9.6 and RHINO are mutually exclusive as demonstrated in the intensity line scan and the inset in the three-color merge image. (**E**) Comparison of R-loop labelling by RHINO and the catalytically dead RNase H1 D210N mutant tagged with a red fluorescent protein (mScarlet-i) in a live U2OS cell co-expressing both proteins. The single confocal z-section images from super-resolved structured illumination microscopy show RHINO spots in the nucleus and nucleolus over a diffuse background, some of which are highlighted by arrowheads in the image and in the intensity line scan. The mScarlet-RNase H1(D210N) shows a diffuse nuclear pattern with some accumulation in the nucleoli but lacking any spot-like accumulations or specific labelling, also demonstrated in the intensity line scan. (**F**) γH2AX immunofluorescence images. Maximum intensity projections from confocal 3D image stacks of formaldehyde-fixed U2OS cells expressing RHINO and of non-transfected (control) cells. The graph shows the quantification of the number of γH2AX spots in RHINO transfected and control cells. Data are not significantly different (n.s.) between the two groups. The number of cells analyzed for each condition is indicated above the graph.

RHINO is composed of a tandem array of three HBDs fused to a fluorescent protein reporter that allows real-time imaging of R-loop foci in live cells (Figure 1A). Imaging of live human osteosarcoma (U2OS) cells and human embryonic kidney cells (HEK293) transiently expressing RHINO and a fluorescent histone H2B protein (mCherry-H2B) revealed exclusively nuclear staining with a strong nucleolar definition and discrete foci of heterogeneous dimensions over a low diffuse background (inset) (Figure 1B, C and Supplementary Movie 1).

Confocal fluorescence microscopy of RHINO-expressing U2OS cells using the S9.6 antibody revealed prominent cytoplasmic staining, which is known to predominantly arise from dsRNA binding (3) (Figure 1D). In contrast, RHINO showed focal accumulations inside nucleoli and throughout the nucleus with reduced co-localization with S9.6 signal, highlighting its superior specificity towards R-loops (Figure 1D). A comparison of different experimental protocols revealed that formaldehyde fixation resulted in a loss of the diffuse nucleolar accumulation of RHINO and intense cytoplasmic staining using the S9.6 antibody (Supplementary Figure 1A). In contrast, both RHINO and S9.6 labelling was best preserved in methanol fixed cells (Supplementary Figure 1A). Methanol followed by acetone fixation preserved the nuclear signal of RHINO but resulted in low nuclear and intense cytoplasmic labelling by S9.6 (Supplementary Figure 1A).

We then co-transfected RHINO and a catalytically inactive version of the human RNase H1 carrying the D210N mutation (D210N) tagged with a red fluorescent protein (23) (Figure 1E). RNase H1 (D210N) showed a diffuse nuclear staining with strong accumulation in nucleoli but no focal labelling nor significant colocalization with RHINO foci as revealed by the line scan analysis (Figure 1E). We also transfected cells with a single RNase H1 hybrid binding domain fused to GFP (1HBD-GFP) (Supplementary Figure 1B). Imaging of 1HBD-GFP showed diffuse pan-nuclear and cytoplasmic staining with more intense accumulation in nucleoli, contrasting with the nuclear focal labeling obtained with RHINO (Supplementary Figure 1B).

To further investigate RHINO’s specificity towards R-loops, we generated two mutant versions of RHINO carrying either W43A or KK59/60AA amino acid substitutions in the HBDs, which are known to compromise RNA:DNA binding (24). Imaging of U2OS cells expressing each of the two mutant molecules revealed non-specific diffuse staining covering the entire nucleus (Supplementary Figure 1C). The lack of discrete foci formed by any of the mutants, suggests that wild-type (WT) RHINO accumulates specifically at sites containing RNA:DNA hybrids.

Next, we sought to investigate if RHINO has a stabilizing effect on R-loops altering their dynamics and leading to DNA damage, the outcome of the unscheduled formation or impaired resolution of R-loops (1). For that, we assessed the formation of nuclear foci containing phosphorylated histone H2AX (γH2AX) in U2OS cells transfected with RHINO (Figure 1F). Analysis of confocal microscopy data revealed a similar number of γH2AX foci in both control and RHINO-expressing cells, suggesting that RHINO does not interfere with R-loop metabolism and dynamics.

**Figure 2.**
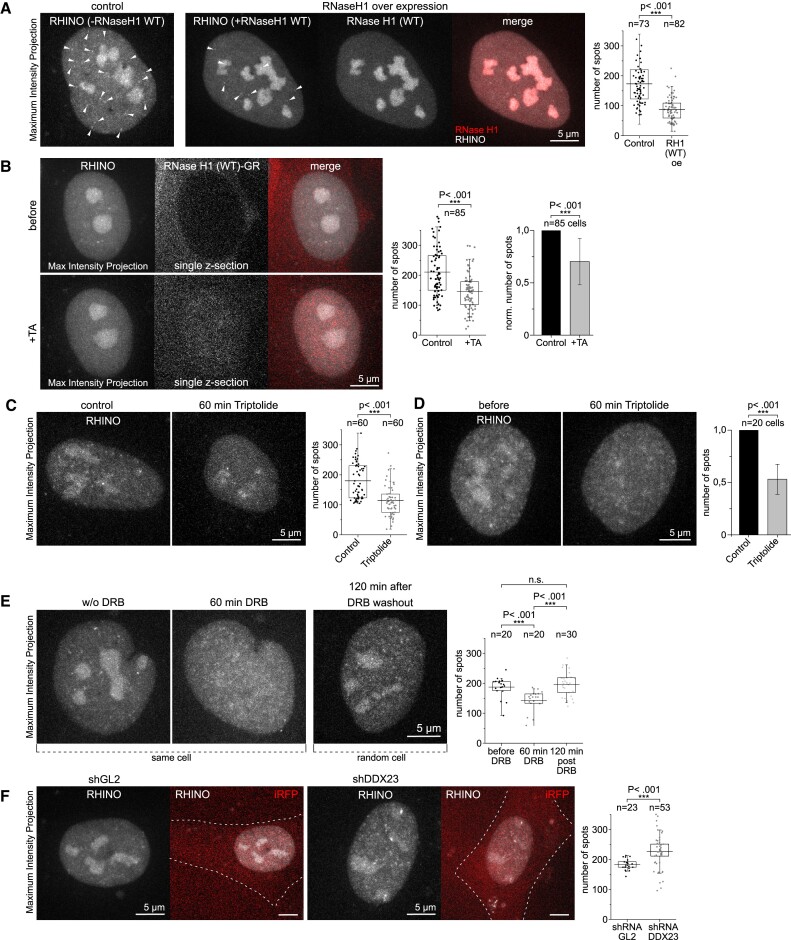
Quantification of R-loops in live cells. (**A**) Maximum intensity projections from spinning disk confocal microscopy 3D image stacks of live U2OS cells expressing RHINO alone (control) or co-expressing human RNase H1 (RNase H1 overexpression) and the quantification of the number of RHINO spots for each condition from three independent experiments. Several RHINO spots are indicated for each condition by white arrowheads to illustrate the readout for the RHINO spot count quantification. (**B**) Imaging and quantification of RHINO spots before and 60 min after (+TA) the nuclear translocation of RNase H1. The graphs show the absolute quantification of detected RHINO spots (dot/box-whisker plot) and the normalized reduction of RHINO spots after 60 min of TA-induced nuclear translocation of RNase H1 from four independent experiments. (**C**) Maximum intensity projections and quantification of RHINO spots in U2OS cells after 60 min of incubation with Triptolide. The number of cells analyzed in each condition is shown above the graph. (**D**) Imaging and quantification of RHINO spots in 20 individual U2OS cells chased for 60 min upon Triptolide treatment. (**E**) Imaging and quantification of RHINO foci in individual U2OS cells chased for 60 min upon DRB treatment, followed by imaging of random cells after DRB washout and a 120-min recovery period. The number of cells analyzed in each condition is shown above the graph. (**F**) Imaging and quantification of RHINO spots in live U2OS cells 24 h after transfection of shRNAs targeting GL2 (control) or DDX23. Data are representative of three independent experiments. The number of RHINO foci detected in each condition is shown in the graph. The number of cells analyzed in each condition is shown above the graph.

To further evaluate the specificity of RHINO, we overexpressed RNase H1 in U2OS cells. Supporting the specific binding of RHINO to RNA:DNA hybrids, we observed a 50% decrease in the number of R-loop foci in RNase H1-overexpressing cells (Figure 2A and Supplementary Table 1). RHINO foci quantified in our analyses are marked by arrowheads (Figure 2A) and magnified in a single confocal z-section (Supplementary Figure 1D). A similar result was obtained upon the acute digestion of R-loops by a glucocorticoid receptor (GR)-fused RNase H1 (RNase H1-GR) (Figure 2B). Addition of the GR-ligand triamcinolone acetonide (TA) to cells expressing RNase H1-GR drove the translocation of RNase H1-GR into the nucleus and significantly reduced the number of R-loop foci within one hour of treatment (Figure 2B). This result is confirmed by overlaying the R-loop signals on single consecutive confocal z-sections and on the corresponding maximum intensity projection of control and TA-treated cells (Supplementary Figure 1E). The reduction in signal, as opposed to a complete loss, was also observed in previous sequencing-based studies (25,26), and is likely due to incomplete digestion of R-loops and to a subset of R-loops that are resistant to RNase H activity (27).

Since R-loops are largely formed during transcription, we sought to test the specificity of RHINO using the transcription inhibitor triptolide. To prevent interference with the protein levels of RHINO, we treated U2OS cells with 1 μM triptolide for just one hour, a time-point that reduces but does not eliminate ongoing transcription and RNA:DNA hybrids (25,28,29). This short treatment was sufficient to significantly decrease the number of R-loop foci detected by RHINO in the bulk cell population (Figure 2C and Supplementary Table 1). To follow the effect of triptolide in individual cells, we imaged the same RHINO-expressing cell before and one hour after triptolide treatment. This experiment revealed a significant 47% reduction in the number of R-loop foci detected one hour after treatment (Figure 2D and Supplementary Table 1).

Subsequently, we quantified R-loop levels in cells subjected to treatment with the reversible transcription inhibitor 5,6-dichlorobenzimidazole 1-β-d-ribofuranoside (DRB) (30). Notably, a significant decrease in the count of R-loop foci identified by RHINO was observed in cells exposed to DRB for a duration of 60 min (Figure 2E). Drug removal and a recovery period of 120 min in fresh medium rescued the R-loop levels to those observed in untreated cells (Figure 2E and Supplementary Table 1).

In a previous study (31), we established that cells lacking the DDX23 helicase exhibit elevated R-loop levels. Here, through 3D imaging and quantification of RHINO foci in *DDX23*-depleted U2OS cells, we observed significantly increased R-loop levels in comparison to control cells (Figure 2F, Supplementary Figure 1E and Supplementary Table 1). Collectively, these findings underscore the specificity of RHINO and establish its competence to use as a reliable tool for quantifying variations in the abundance of R-loops resulting from perturbations in factors involved in their processing.

### R-loop dynamics in nuclear substructures

**Figure 3.**
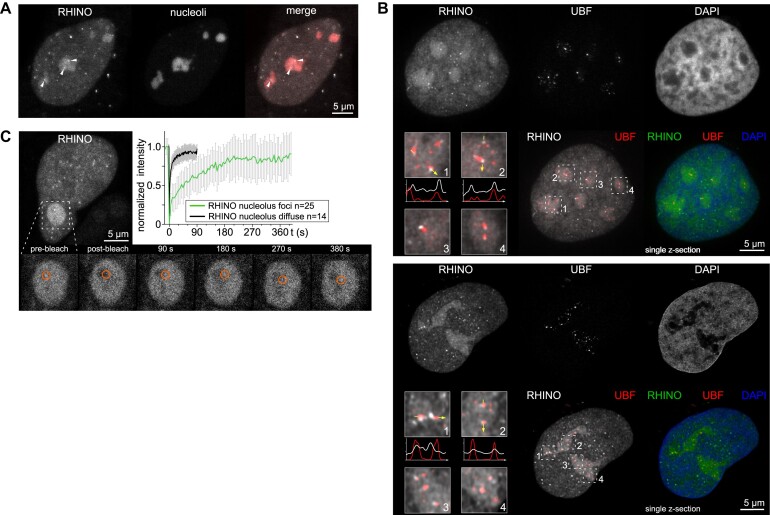
R-loop dynamics in nucleoli. (**A**) Imaging of R-loops and a nucleolar marker in live U2OS cells expressing RHINO. Individual RHINO spots in two nucleoli are indicated by arrowheads. (**B**) Imaging of RHINO-expressing cells fixed and stained with an anti-UBF antibody. Display of two super-resolution microscopy image sets acquired with structured illumination (top) and Airyscan 2 technology (bottom). The three-colour merge image shows a single confocal z-section from a 3D z-stacks covering the entire nuclear volume. The corresponding maximum intensity projections are shown for the individual channels and the RHINO/UBF merge image. The magnified inset images and intensity line scans show the colocalization of nucleolar R-loops labelled by RHINO and UBF. (**C**) FRAP of nucleolar R-loops revealed by RHINO foci and of diffuse nucleolar RHINO in U2OS cells. The fluorescence within the red circle targeting a RHINO focus was bleached (magnified time series images) and its recovery was monitored over time. FRAP curves display the mean fluorescence intensity (±SD) of RHINO foci from 25 cells and of diffuse nucleolar RHINO from 14 cells.

Owing to the lack of robust tools to image and collect quantitative parameters of R-loops in live cells, our understanding of R-loop dynamics and of their specific kinetics at distinct genomic loci is scarce. Nucleoli are nuclear structures where R-loops are abundant (32). In agreement, strong nucleolar staining was observed in cells expressing RHINO, including discrete foci (Figure 3A). Notably, the nucleolar foci co-localized with RNA Polymerase I transcription factor upstream binding factor (UBF) foci (Figure 3B). UBF labels sites of active ribosomal RNA transcription at the periphery of the fibrillar centres and in the dense fibrillar component of nucleoli, where R-loops are abundant (33). To investigate the dynamics of nucleolar R-loops, we performed fluorescence recovery after photobleaching (FRAP) experiments in individual nucleolar RHINO foci (Figure 3C and suppl. Movie 2). We observed that nucleolar RHINO foci restored 50% and 80% of the initial fluorescence intensity in 25 and 135 s, respectively (Figure 3C and Table 1). As a control, we measured the dynamics of diffuse nucleolar RHINO molecules not bound to R-loops, which exhibited even faster dynamics, with 50% fluorescence restoration occurring within 2 s and 80% within 8 s (Table 1). The observed kinetics of nucleolar R-loops is comparable to that of the elongation phase of RNA polymerase I on ribosomal genes, which takes up to 182 s to complete a transcription cycle (34). Altogether, these data suggest that R-loops are formed and resolved during consecutive cycles of ribosomal DNA transcritpion and illustrate the capacity of RHINO to provide kinetic parameters of these structures at specific nuclear regions in live cells.

**Table 1.** Summary of the kinetic parameters obtained in the FRAP experiments performed at nucleolar and telomeric R-loops

	**Nucleoli**		**Telomeres**				
	**RHINO foci**	**Diffuse RHINO**	**RHINO ‘FAST’**	**RHINO ‘SLOW’**	**RHINO W43A**	**RHINO KK59/60AA**	**GFP**
**Number of FRAP experiments**	25	14	37	22	16	13	8
**Time to 80% FRAP**	135 s	8 s	8.1 s	40 s	2.4 s	2.1 s	2.4 s
**Time to 50% FRAP**	25 s	2 s	1.5 s	4 s	0.6 s	0.6 s	0.8 s
**Fold over diff./mut. RHINO 80%**	16.875	-	3.6	17.78	-	-	-
**Fold over diff./mut. RHINO 50%**	12.5	-	2.5	6.67	-	-	-

**Figure 4.**
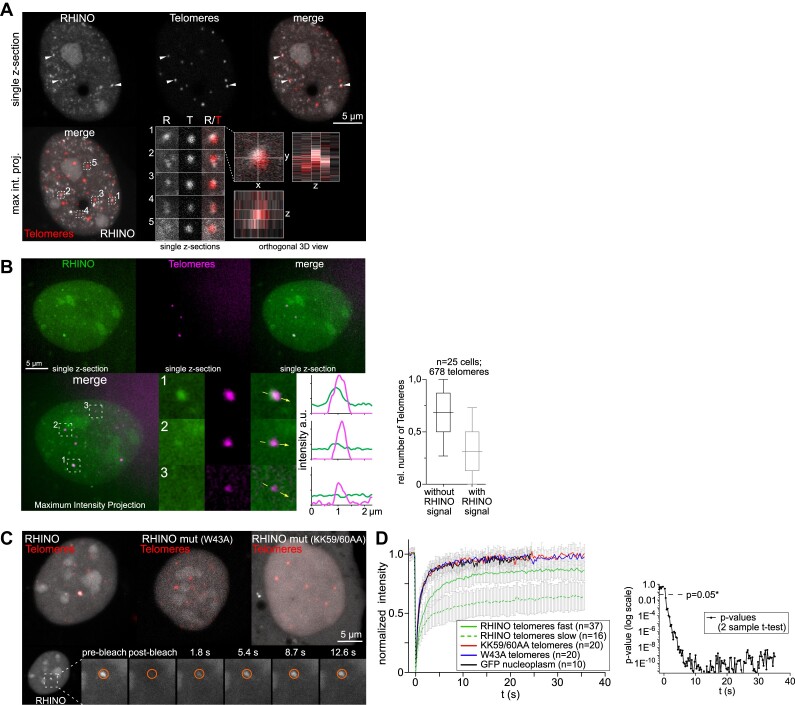
R-loop dynamics in telomeres. (**A**) Imaging of live U2OS cells co-expressing RHINO and iRFP-TRF2 to label telomeres. The magnified inset images from single confocal z-sections show individual telomeres (T) with colocalizing RHINO (R) signals. An orthogonal 3D view of the magnified inset region 1 shows the colocalization of R-loops and TRF2. (**B**) Live cell super-resolution structured illumination microscopy of RHINO and telomeres to visualize telomeric R-loops. The insets show single z-sections of individual telomeres with colocalizing RHINO signals of different intensities (1 )and (2) as well as a telomere without RHINO signal above the background level (3). The intensity line scans demonstrate the different levels of RHINO labelling for the telomeres in the magnified insets. The graph shows a quantification of the number of telomeres with RHINO fluorescence intensity above the diffuse background intensity from 3D spinning disk confocal microscopy images from three independent experiments. (**C**) FRAP of telomeric R-loops detected with RHINO and FRAP of the RHINO mutants at telomeres to serve as controls. The magnified region shows the RHINO fluorescence intensity at the bleached region (red circle) over time. (**D**) FRAP curves of RHINO and RHINO mutants (KK59/60AA and W43A) at telomeres and for free diffusing GFP molecules in the nucleoplasm (GFP) from four independent experiments. Curves display the mean fluorescence intensity (±SD). The *P*-value curve is derived from 2-sample *t*-tests comparing each time point of the faster and slower recovering fractions (solid and dashed green FRAP curve, respectively) of RHINO at telomeres.

Telomerase-negative cancer cells that rely on the alternative lengthening of telomeres (ALT) mechanism for telomere elongation (35) are characterized by high levels of telomeric R-loops (36). U2OS are amongst the ALT cells where telomeric R-loops are abundant (36). In agreement, several foci observed in U2OS cells expressing RHINO co-localized with TRF2 foci, a component of the shelterin complex that assembles at telomeres (Figure 4A and Supplementary Movie 3). This finding was further validated using super-resolution imaging (structured illumination) of RHINO and TRF2 in live cells. This experiment detected R-loops in 32% of all telomeres (Figure 4B and Supplementary Table 1). To measure the dynamics of telomeric R-loops, we performed FRAP experiments in cells expressing the WT or mutant versions of RHINO (Figure 4C). Notably, these experiments revealed two distinct populations of telomeric R-loops: a major fraction (70% of all R-loops analyzed) in which the fluorescence recovered to 80% of its initial intensity within 8.1 s after photobleaching (solid green line), and another, significantly slower, which recovered to a maximum of 65% of its initial fluorescence intensity after 35 s (dashed green line) (Figure 4D, Table 1 and Supplementary Movies 4 and 5). The faster fraction recovered 50% of the initial fluorescence intensity in 1.5 s, whereas the slower fraction took 4 s to recover the same percentage of fluorescence. The two curves are significantly different, representing two telomeric R-loop fractions with distinct dynamics (Figure 4D). In a supplementary set of FRAP experiments with longer time intervals between image acquisitions (2 s) and a total duration of 126 s (Supplementary Figure 2A), we tracked the slower recovering fraction of R-loops until they reached the fluorescence recovery plateau. The slower fraction of telomeric R-loops took 40 s to restore 80% of the initial fluorescence, a recovery rate about 5 times slower compared to the faster fraction (Table 1). FRAP experiments on cells expressing the W43A and KK59/60AA RHINO mutants or GFP alone, revealed a much faster fluorescence recovery that was determined by the diffusion rate of the proteins (Figure 4D), suggesting that the WT RHINO FRAP is determined by its specific binding to telomeric R-loops. These data disclose the existence of two subpopulations of telomeric R-loops: a more frequent and labile one and a fraction of more persistent R-loops. The former likely corresponds to co-transcriptional R-loops formed and rapidly resolved by repetitive telomeric transcription cycles. R-loops may also be formed in *trans*, including in non-transcribed telomeres (37), originating structures that are not continuously replaced by successive transcription cycles and may constitute the slower fraction of telomeric R-loops.

### Live-cell imaging of R-loops at an actively transcribed gene

**Figure 5.**
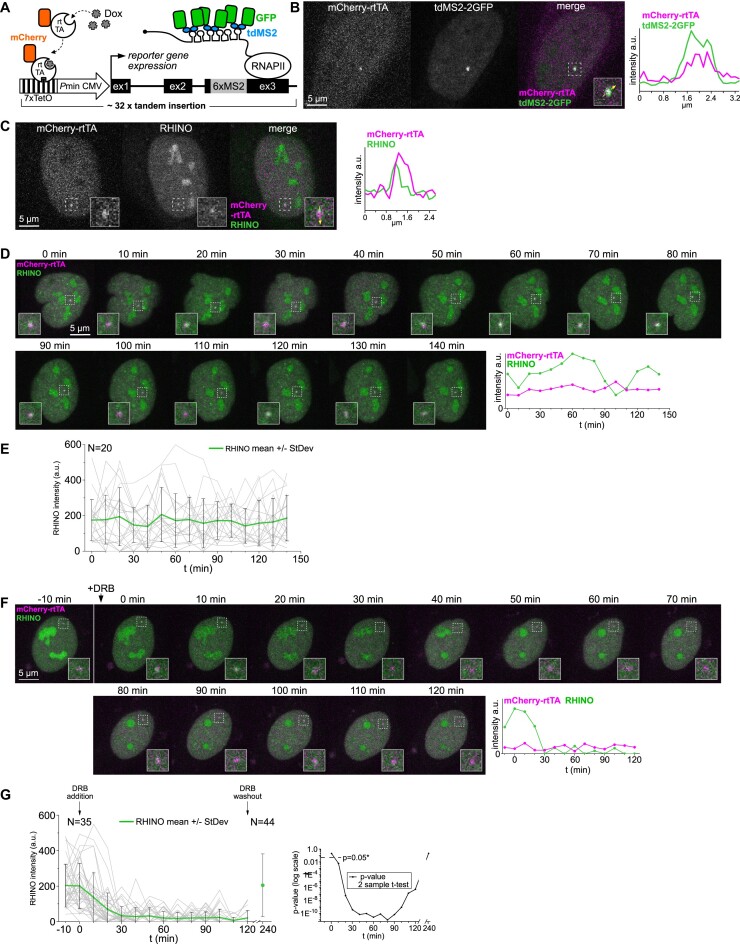
Live-cell imaging of R-loops at an actively transcribed reporter gene. (**A**) Graphic representation of the *β-*globin reporter gene. (**B**) Colocalization of mCherry-rtTA and MS2-GFP signals upon doxycycline addition in U2OS cells. The intensity line scan shows overlapped fluorescence signals. (**C**) Imaging of R-loops formed at the *β*-globin reporter gene array in live U2OS cells expressing RHINO. The graph shows the fluorescence intensity along the line (inset). (**D**) Single confocal z-section images of a U2OS cell expressing RHINO to label R-loops and mCherry-rtTA to label the *β*-globin reporter gene array. The cell was imaged over 140 min at 10-min intervals and the corresponding graph shows the R-loop quantification (RHINO: green) at the centre of intensity of the mCherry-rtTA spot (magenta). (**E**) The graphs represent the R-loop quantification by RHINO fluorescence intensity measurements at labelled *β*-globin reporter gene loci as in d) for 20 individual cells and the mean RHINO fluorescence intensity (solid green ± SD). (**F**) Single confocal z-section images of a U2OS cell expressing RHINO to label R-loops and mCherry-rtTA to label the *β*-globin reporter gene array. The cell was imaged before and chased after DRB addition to the growth medium at 10-min intervals for a total of 120 min. The corresponding graph shows the R-loop quantification (RHINO: green) at the centre of intensity of the mCherry-rtTA spot (magenta). (**G**) The graphs represent the R-loop quantification by RHINO fluorescence intensity measurements at labelled *β*-globin reporter gene loci of 35 cells from three independent experiments and the mean values (solid green line, ±SD). The cells were each imaged before and chased upon DRB incubation as in e). After 120 min of DRB incubation, the cells were washed, supplied with fresh growth medium without DRB and allowed to recover for 120 min before quantification of RHINO fluorescence at the reporter gene locus. The *P*-value graph is derived from 2-sample *t*-tests comparing the mean RHINO intensity values from the time point before DRB addition to each time point after DRB addition as well as after DRB washout and recovery.

Most described physiological roles of R-loops relate to their impact on protein-coding gene transcription by RNA polymerase II (2). Yet, the lack of tools to properly detect and monitor these structures in live cells precluded a deeper understanding of the mechanisms and factors governing such functions. Here, we sought to gather novel kinetic parameters of R-loops formed at protein-coding genes in human cells. For that, we established a cell line containing a tandem genomic integration of a human *β-globin* reporter gene array carrying the MS2 system to image transcription *in vivo* (38) (Figure 5A). To avoid MS2-binding protein (MS2CP) interference with R-loop formation (39), we tagged a reverse tetracycline transactivator (rtTA) with a red fluorescent protein (mCherry) to simultaneously label the reporter gene locus and induce its transcription upon doxycycline (Dox) addition (Figure 5A, B). R-loops formed at the reporter gene were imaged in live cells expressing RHINO and mCherry-rtTA, but not MS2CP, upon the addition of Dox (Figure 5C). A 140 min-long live-cell 3D imaging at 10 min intervals provided unique recordings of the real-time fluctuations of R-loop levels during consecutive transcription cycles (Figure 5D). Imaging of cells over 140 min revealed highly asynchronous RHINO signal fluctuations at the transcribed reporter gene (Figure 5E). Imaging RHINO at the reporter gene with a higher temporal resolution of 10 s revealed R-loop formation and suppression cycles that did not follow a regular period (Supplementary Figure 2B). We then treated cells with the reversible transcription inhibitor DRB and followed R-loop dynamics at the reporter gene. We measured the fluorescence intensity of RHINO at the reporter gene locus of DRB-treated cells over 120 min at 10 min intervals (Figure 5F). RHINO signal was lost approximately 30 min after DRB treatment and persisted at background levels for the entire duration of the experiment. Imaging RHINO with a higher temporal resolution of 10 s showed only very low-intensity stochastic fluorescence signals after DRB treatment (Supplementary Figure 2C). Quantification of the RHINO fluorescence intensity at the reporter gene locus of 35 cells before and after DRB treatment revealed that DRB addition results in a highly significant decrease to background values that is indicative of a robust depletion of R-loops concurrent with transcription inhibition (Figure 5G). We then washed out the DRB-containing medium and allowed cells to recover in fresh Doxycycline-containing growth medium for 120 min. Due to the loss of the original cell positions under the microscope during the DRB washing-out procedure, we imaged 44 random cells to measure RHINO fluorescence intensity at the reporter gene locus (Figure 5G). In agreement with the reversibility of the DRB-mediated transcriptional inhibition, drug washout increased RHINO fluorescence intensity at the reporter gene to pre-treatment values (Figure 5G). To assess the statistical significance of the DRB effect on RHINO signal, we performed a 2-value-*t*-test comparing the RHINO fluorescence intensity before DRB addition to that observed at each time point after DRB treatment and plotted the *p*-values in a graph (Figure 5G). A statistically significant reduction of RHINO fluorescence was observed 10 min after DRB addition and persisted until DRB washout. Recovery of transcription for 120 min after DRB washout restored the RHINO fluorescence levels of non-treated cells (Figure 5G and Supplementary Figure 2D).

**Figure 6.**
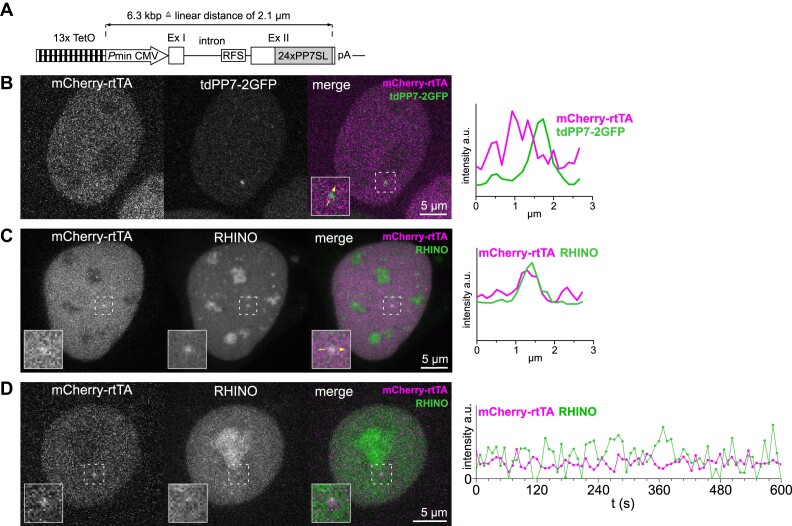
Live-cell imaging of R-loops at a single transcribed gene. (**A**) Illustration of the IgM reporter gene containing an R-loop forming sequence (RFS) and an array of 24 PP7 stem-loops (24xPP7SL). The reporter gene was integrated as a single copy into the genome of U2OS cells. (**B**) Expression of mCherry-rtTA reveals the location of the single reporter gene locus and the co-expressed GFP-PP7 reveals its transcriptional activity in a single confocal z-section. The intensity line scan shows the juxtaposed labelling of the reporter gene and labelled transcripts, which is a consequence of the distance between the labelling sequences in the reporter gene. (**C**) Imaging of R-loops at the actively transcribed reporter gene locus. A single confocal z-section of a U2OS reporter gene cell co-expressing RHINO and mCherry-rtTA shows the R-loop labelling at the reporter gene locus, also featured in the magnified insets and in the intensity line scan. (**D**) Time-series imaging of RHINO at the IgM reporter gene locus shown in the single confocal z-section images and magnified insets. The graph shows the RHINO fluorescence intensity fluctuations measured at the transcriptionally active mCherry-rtTA-labelled reporter gene locus.

To image R-loops formed during the transcription of a single gene, we inserted the R-loop-prone sequence found in the *β*-*actin* gene (40) within the intron of the mouse IgM reporter gene, also containing PP7 stem-loops in exon II to directly visualize its transcription (Figure 6A) (8). Furthermore, to enhance the signal-to-noise ratio of the reporter gene locus labelling, we introduced a total of 13 Tet operator (TetO) repeat sequences, thereby increasing the number of binding sites for mCherry-rtTA molecules. The intensity line scans show the overlap of mCherry-rtTA with PP7-GFP (Figure 6B) and RHINO (Figure 6C) and validate the competency of RHINO to detect R-loops formed on individual active genes with high sensitivity and spatial resolution. The offset of the mCherry-rtTA and PP7-GFP signal peaks (Figure 6B) illustrate the distance between the promoter TetO labelling sequences and the full-length PP7 stem-loop array of the nascent pre-mRNA molecules (Figure 6A). For enhanced temporal resolution in measuring R-loop dynamics at a single active gene, we conducted 3D imaging of RHINO at the IgM reporter over a 10 min period with 8-second intervals between image acquisitions (Figure 6D and Supplementary Movie 6). These data showed stable mCherry-rtTA reporter gene labelling and RHINO fluctuation cycles lasting approximately 30 to 60 s. This time interval is consistent with the expected duration of reporter gene transcription, estimated to take around 90 s to transcribe the 6.3 kb at a transcription rate of 4kb/min (8,41). Altogether, these findings reveal the remarkable sensitivity of RHINO and showcase its potential for utilization in studies focused on probing the dynamics of R-loops at single genes.

## Discussion

In this study, we describe RHINO, a genetically encoded sensor that selectively binds RNA:DNA hybrids for live-cell imaging of cellular R-loops with unprecedented resolution and high specificity and sensitivity. This tool enables the quantification of R-loop distribution and dynamics in live cells. RHINO stands as a versatile tool that can be readily expressed across a wide range of cell types and visualized through conventional confocal, spinning disk, or advanced super-resolution microscopy techniques like Airyscan or structured illumination. Although not explored here, RHINO can be subject to modifications through the incorporation of suitable tags, rendering it amenable for utilization in pulldown-based assays, including genome-wide R-loop profiling techniques like chromatin immunoprecipitation followed by sequencing (ChIP-seq). Our data reveal RHINO’s superior R-loop labelling when compared to the S9.6 antibody and the catalytically inactive RNase H1 enzyme. Confocal microscopy imaging of RHINO showed exclusively nuclear staining characterized by discrete foci that colocalize with active genes, telomeric and nucleolar factors and are significantly suppressed upon transcription inhibition. Although the formation of R-loops within mitochondria has been established (42), it's important to note that RHINO's inability to localize to mitochondria due to the absence of a mitochondrial targeting signal restricts its capability to label this particular subset of R-loops. Expression of mutant variants of RHINO that have lost their RNA:DNA binding capacity led to nonspecific and diffuse nuclear staining, akin to the pattern observed when R-loops are digested through RNase H1 overexpression. Our results confirm that the foci detected by RHINO correspond to R-loop locations and underscore the robust specificity of this genetically encoded sensor. Importantly, the absence of increased DNA damage, particularly DNA double-strand breaks, in cells expressing RHINO strongly suggests that RHINO does not interfere with R-loop dynamics. This is significant considering that the stabilization of R-loops in cells lacking the activity of helicases, like DDX23, or RNase H enzymes typically results in the accumulation of DNA damage (31), a phenotype not observed in cells expressing RHINO.

We measured R-loop dynamics at specific genomic loci—nucleoli, telomeres and protein-coding genes—enabling the extraction of unprecedented parameters related to these nucleic acid structures. In the nucleoli, the observed kinetics of R-loop formation suggest that these structures are generated and resolved during consecutive cycles of RNA polymerase I transcription of rDNA genes. The analysis of R-loop dynamics at the telomeres revealed the presence of two distinct classes of R-loops: one, more frequent, characterized by a more transient nature and another exhibiting greater persistence. The former may represent co-transcriptional R-loops, which arise and resolve rapidly during repetitive telomeric transcription cycles. On the other hand, R-loops can also form in trans (37), even at non-transcribed telomeres, a process that may originate structures that are not continuously replaced by ongoing telomeric transcription cycles and may correspond to the persistent class of telomeric R-loops observed in this study. When corrected against the kinetics of protein diffusion in each locus, the fraction of fast telomeric R-loops showed the highest turnover rates. Nucleolar R-loops and the slower fraction of telomeric R-loops displayed comparable and slower dynamics, particularly when evaluating the time needed to achieve 80% fluorescence recovery in FRAP experiments (as summarized in Table 1, data expressed as fold change over diffuse/mutant RHINO 80%).

To investigate R-loop dynamics at an active gene locus, we used the reversible transcription inhibitor DRB. Upon DRB treatment, RHINO labelling at the reporter gene locus was rapidly lost, indicative of R-loop depletion following transcription inhibition. The original RHINO fluorescence intensity observed at the reporter gene locus was completely restored after the drug washout. These results underscore the plasticity of R-loop formation and resolution and their responsiveness to transcriptional changes, highlighting the competency of RHINO to collect accurate kinetic measurements of R-loops at different genomic loci. Our findings further demonstrated the high sensitivity of RHINO and established its capacity to be used as an R-loop sensor in studies aimed at investigating the dynamics of these structures at individual genes. Overall, our study reveals the heterogeneity of R-loop dynamics and discloses the existence of distinct R-loop populations present at telomeres. The ability of RHINO to collect accurate kinetic data from live cells affords unique opportunities for future studies aiming at delineating their roles in processes such as transcription, telomere maintenance, ribosomal RNA biogenesis or the other functions on which they impinge.

## Supplementary Material

gkad812_Supplemental_Files

## Data Availability

The data underlying this article are available in the article and in its online supplementary material.
