# Supplementary material for: Live-cell imaging unveils distinct R-loop populations with heterogeneous dynamics
Source: Nucleic Acids Res. 2023 Oct 11;51(20):11010–23. doi: 10.1093/nar/gkad812 (PMC10639055; doi:10.1093/nar/gkad812)
Supplement: gkad812_Supplemental_Files [file gkad812_supplemental_files.zip › suppl Table 1.pdf]

	RHINO spot number quantification - random cells in bulk population				
	control (cells w/o RH1 oe)	RNaseH1 over expression		control (no Triptolide)	60 min Triptolide
sample size	n = 73	n = 82		n = 60	n = 60
mean (nr. of foci)	163,4	87,4		179,833	114,75
%	**100**	**53,5**		**100,0**	**63,8**

	RHINO spot number quantification - same cells before and after treatment				
	before TA (no RH1-GR induction)	60 min TA (+RNase H1-GR)		before Triptolide	60 min Triptolide
sample size	n = 85	n = 85		n = 20	n = 20
mean (nr. of foci)	206,6	142,6		193,4	103,85
%	**100**	**69,0**		**100**	**53,7**

	RHINO spot number quantification		
	same cells before and after DRB		random cells in population
	before DRB	60 min DRB	after DRB removal + 120 min recovery
sample size	n = 20	n = 20	n = 30
mean (nr. of foci)	186,8	143,05	196,633
%	**100**	**76,6**	**105,3**

	RHINO spot number quantification random cells in population	
	shGL2	shDDX23
sample size	n = 23	n = 53
mean (nr. of foci)	182,8	226,1
%	**100**	**123,7**

	Telomeres with RHINO signals	Telomeres without RHINO signals
sample size	n = 25 cells	n = 25 cells
%	**31,6**	**68,4**
