# Supplementary material for: Live-cell imaging unveils distinct R-loop populations with heterogeneous dynamics
Source: Nucleic Acids Res. 2023 Oct 11;51(20):11010–23. doi: 10.1093/nar/gkad812 (PMC10639055; doi:10.1093/nar/gkad812)
Supplement: gkad812_Supplemental_Files [file gkad812_supplemental_files.zip › supplementary material legends.pdf]

SUPPLEMENTARY FIGURE LEGENDS

Supplementary Figure 1. R-loop labelling, detection, and quantification in live and fixed cells. a) Comparison of different cell fixation protocols using formaldehyde, methanol, or methanol/acetone for RHINO and S9.6 antibody labelling of R-loops in U2OS cells. **b)** Expression of GFP-1HBD and RHINO in U2OS cells to compare the labelling of R-loops by each sensor. The images show single confocal z-sections. The cell nucleus in the GFP-1HBD image is indicated by a dashed line. **c)** Expression of two RHINO mutant proteins in U2OS cells. **d)** Detection of RHINO foci of different sizes and intensities using the STaQTool software to identify and quantify diffraction-limited objects in microscopic images. A single confocal z-section and the corresponding maximum intensity projection of the same cell shown in Fig. 2a are displayed. The RHINO foci annotated in the single z-section and in the magnified regions demonstrate the size and intensity heterogeneity of RHINO labelling of R-loops in live cells. The same positions are annotated in the corresponding maximum-intensity projection image. **e)** Detection and quantification of RHINO foci number using the Spot Detector plugin in the ICY bioimage analysis software package. The imaged cells are the same as in Fig. 2b. with three central single confocal z-sections displayed before (top row) and after 60 minutes of TA addition (bottom row) with the detected RHINO foci annotated as pixel masked in white. Note that some RHINO foci can span several adjacent z-sections if the pixels connect. These foci covering two or more z-sections are registered as a single 3D object (arrows). The corresponding maximum intensity projections from all z-sections with all annotated RHINO foci are shown on the right. The projections contain foci that overlap but originate from separate objects in different z-planes and represent individual foci in the 3D analysis for the spot number quantification. **f)** Western blot analysis showing the reduction of DDX23 protein levels 24 hours after transfection of the shRNA expression plasmids targeting either GL2 (control) or DDX23. α-tubulin serves as a gel-loading control.

Supplementary Figure 2. Live cell R-loops dynamics. a) FRAP experiments to capture the complete dynamics of the slow recovering telomeric R-loop fraction. Curves display the mean fluorescence intensity (±SD) from three independent experiments. **b)** 3D time-series imaging of R-loop dynamics detected with RHINO at

the actively transcribed β-globin reporter gene array. The two images show single confocal z-sections of U2OS cells and the corresponding graphs show the reporter gene labelling by mCherry-rtTA (magenta) and R-loop labelling by RHINO (green) over time. **c)** Imaging setup as in b) for cells incubated with 100 µM DRB for 120 minutes. The reporter gene is labelled by mCherry-rtTA (magenta) and R-loop detected by RHINO (green). **d)** Imaging of a cell after DRB washout and a 120-minute recovery period. The reporter gene and R-loops are labelled by mCherry-rtTA (magenta) and RHINO (green), respectively.

SUPPLEMENTARY TABLE LEGENDS

Supplementary Table 1. Summary of the RHINO quantification data obtained in the different experimental settings described in this study.

SUPPLEMENTARY MOVIES

Supplementary Movie 1. Movie of spinning disk confocal microscopy 3D time-series imaging of RHINO in three U2OS cells. The cells were imaged at 5 minutes intervals over 60 minutes to emphasize the live cell dynamics of R-loops labelled by RHINO.

Supplementary Movie 2. Movie of a RHINO spot FRAP experiment for nucleolar R-loops to demonstrate the dynamics of RHINO fluorescence recovery. The fluorescence was bleached in the indicated circular region of interest and images were acquired at 5 seconds intervals over more than 6 minutes.

Supplementary Movie 3. Movie of a spinning disk confocal microscopy 3D time-series imaging of RHINO and labelled telomeres in a live U2OS cell. The cell was imaged at 3 minutes intervals over 90 minutes to demonstrate the dynamics of R-loops at telomeres with RHINO signal (arrowheads in the first frame).

Supplementary Movie 4. Movie of a FRAP experiment for telomeric R-loops labelled by RHINO with fast recovery dynamics. The fluorescence was bleached in the indicated circular region of interest and images were acquired at 300 milliseconds intervals over 35 seconds.

Supplementary Movie 5. Movie of a FRAP experiment for telomeric R-loops labeled by RHINO with slow recovery dynamics. The fluorescence was bleached in the indicated circular region of interest and images were acquired at 2 seconds intervals over 2 minutes.

Supplementary Movie 6. Movie of a spinning disk confocal microscopy 3D time series imaging of R-loops labeled by RHINO at a single transcribed reporter gene in a live U2OS cell. The cell was imaged at 8 seconds intervals over 10 minutes.
