# Supplementary figures and images for: Live-cell imaging unveils distinct R-loop populations with heterogeneous dynamics
Source: Nucleic Acids Res. 2023 Oct 11;51(20):11010–23. doi: 10.1093/nar/gkad812 (PMC10639055; doi:10.1093/nar/gkad812)

suppl. Figure S1

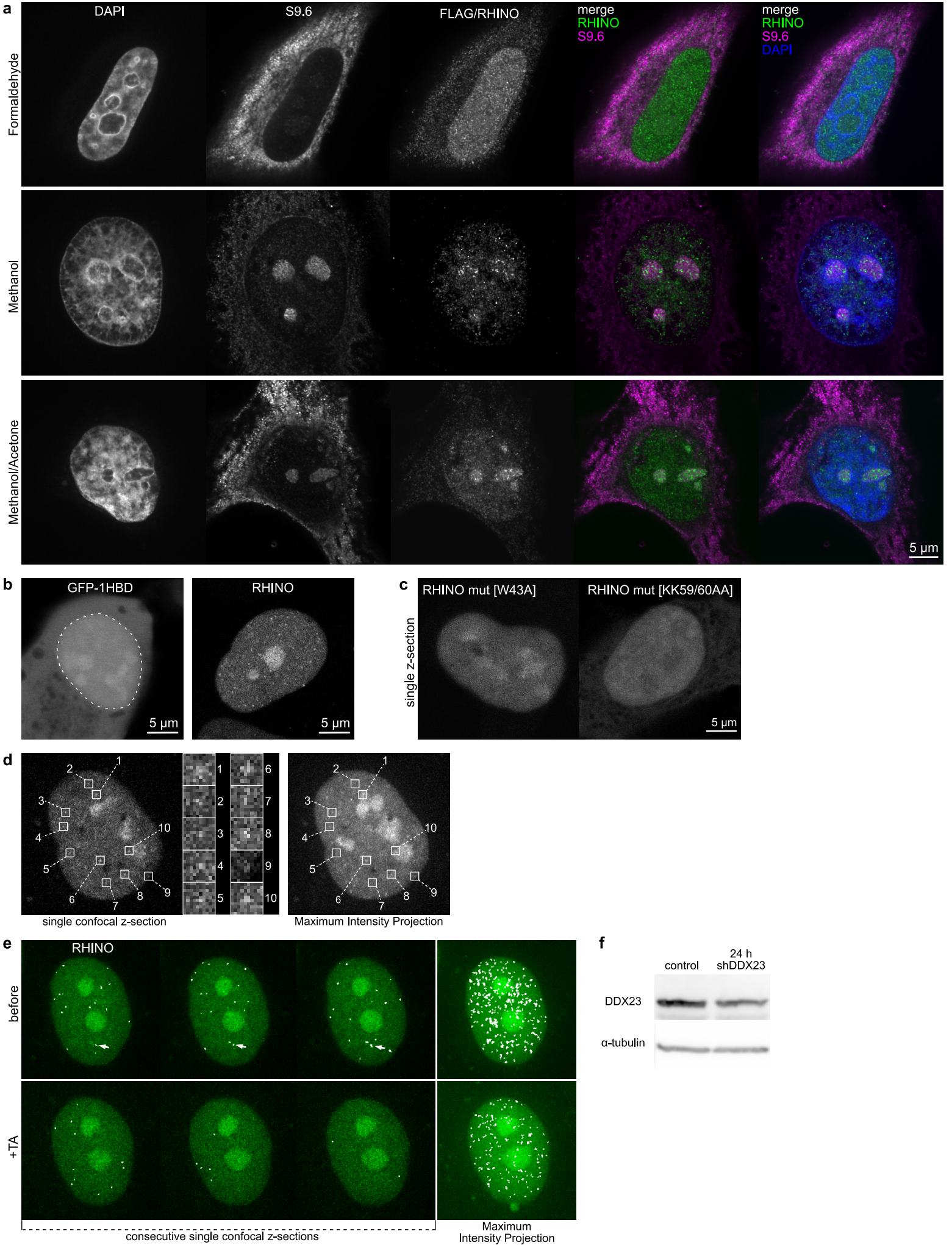

Supplement: gkad812_Supplemental_Files [file gkad812_supplemental_files.zip › Supplementary Figure 1.pdf]

suppl. Figure S2

a

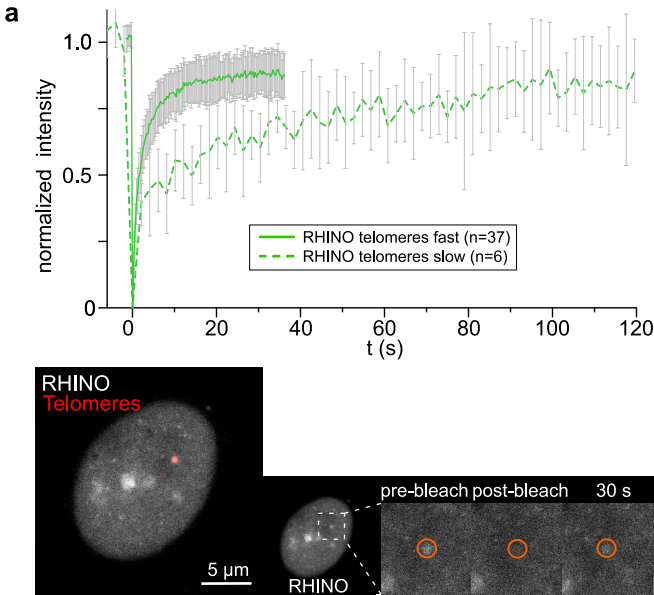

b

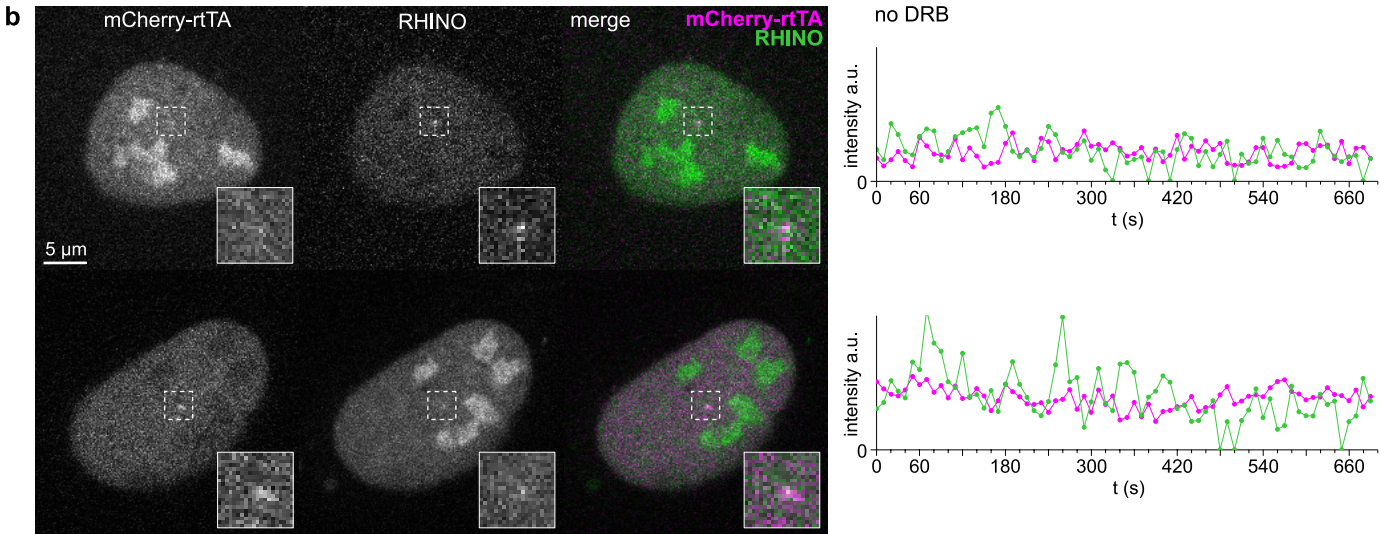

c

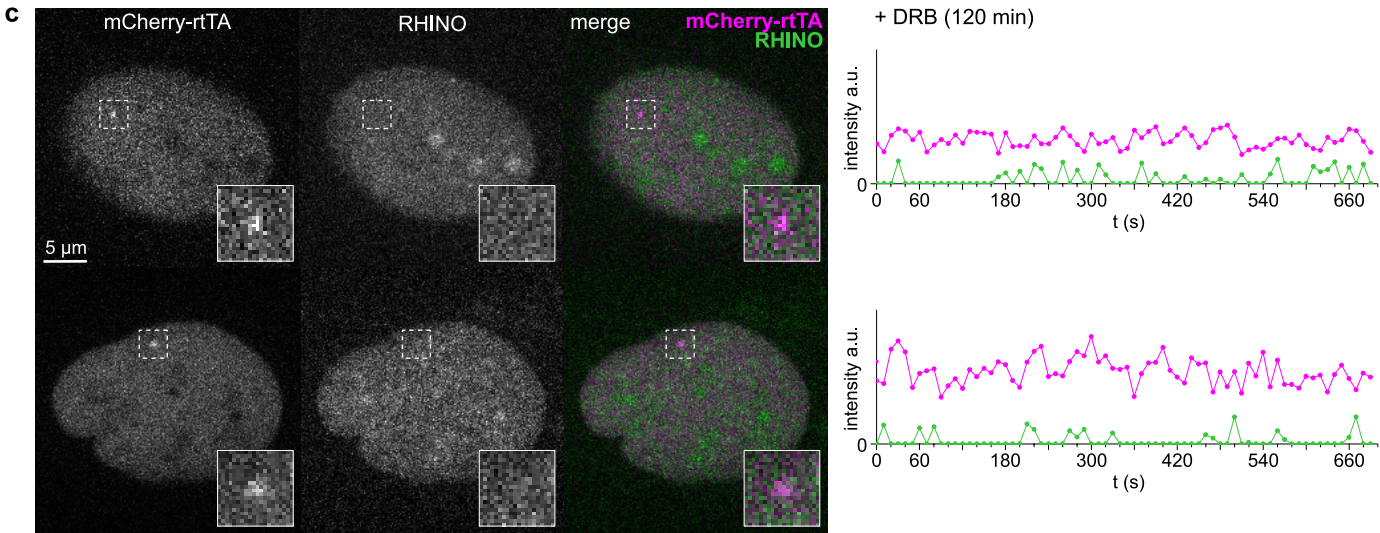

d

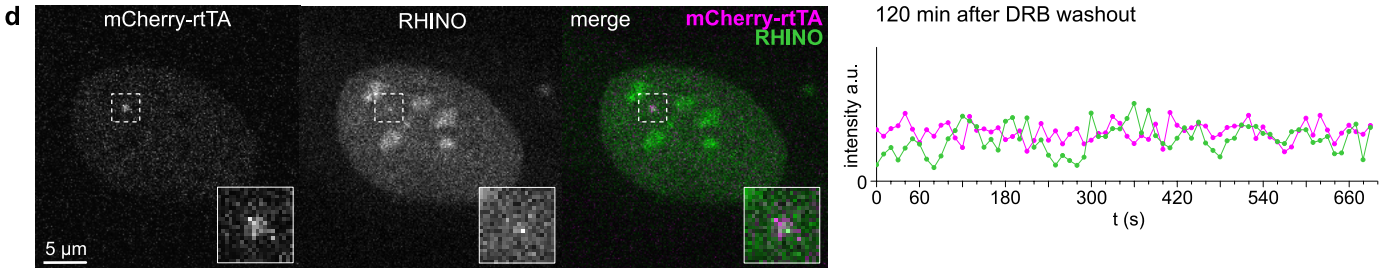

Supplement: gkad812_Supplemental_Files [file gkad812_supplemental_files.zip › Supplementary Figure 2.pdf]
